# Anxiety, eating disorders and menstrual disturbances risk in Spanish elite female paddlers

**DOI:** 10.1038/s41598-024-66167-x

**Published:** 2024-07-02

**Authors:** N. Aquino-Llinares, J. Gavala- González, M. E. Porras-García

**Affiliations:** 1 Department of Economics, Quantitative Methods and Economic History, University of Pablo de Olavide, Carretera de Utrera Km 1, 41013 Seville, Spain; 2 Department of Physical Education and Sports, University of Seville, 41013 Seville, Spain; 3 Department of Physiology, Anatomy and Cellular Biology, University of Pablo de Olavide, Carretera de Utrera Km 1, 41013 Seville, Spain

**Keywords:** Neurological disorders, Nutrition disorders

## Abstract

Affiliated athletes are frequently subject to higher levels of anxiety due to the intensity of training, competition and many other factors. This anxiety can cause alterations in their health, both physically and mentally, such as menstrual irregularities, eating disorders, etc. In this work we have analysed the anxiety levels of a population of female affiliated paddlers and the possible consequences for their health. The results showed that a third of the female paddlers analysed have a moderate/high risk of suffering from anxiety; and within this group, the less sporting experience the athlete has, the greater the probability is of suffering from anxiety. Moreover, almost half the total of the female paddlers suffers from menstrual dysfunction before an important competition, with this number rising among high performance athletes, even though three out of every four adjust their training schedule to their menstrual cycle. Less-experienced female athletes, who show higher anxiety levels, also present a greater risk of suffering from eating disorders.

## Introduction

Within the Spanish sport model, affiliated athletes (who compete in different competitions organised by sports federations) may be High Performance Athletes (HPA) and High Level Athletes (HLA). The HLA are those whose performance and classification place them among the best in Europe or in the world. This status is achieved as a result of their participation in competitions organised by the international federations that regulate each sport or by the International Olympic Committee. These athletes appear on several lists that are published on an annual basis by the *Consejo Superior de Deportes* (Spanish National Sports Council). In contrast, the HPA do not obtain such notable results but they undoubtedly deserve to be distinguished from other athletes due to their achievements. Of all the sports practised in Spain, canoeing is currently the most successful worldwide; it is the second national sport with the most Olympic medals. In the last World Championships in Canada, Spain was the country that won the most medals with a total of eight, three of them won by women.

However, practising sport at a high level can place great pressure on elite athletes, especially on women. The diagnosis of depressive disorders and anxiety is approximately twice as common among women than among men^1^ and women suffer from eating disorders between six and ten times more frequently than men^2–4^. High anxiety levels can affect the brain’s attention networks and, therefore, its executive function, the processing of stimuli and the selection of information^5^, all of which are fundamental in elite sport^6^. Situations involving stress, such as intense sports training sessions, can also affect a regular menstrual cycle, which is associated with being in good physical and mental condition. Many authors have described anxiety disorders in elite female rowers/paddlers and have linked them to menstrual irregularities, such as delayed onset of puberty, amenorrhea and premature osteoporosis^7,8^. Although it has been observed that sport in general can cause certain menstrual disorders in women, there is some controversy about the type and intensity of physical activity^7,9,10^. These alterations in athletes have also been linked to short sleep duration^11^, low levels of vitamin D^9,12^ or lack of energy^7,13^. The relationship between anxiety levels and menstrual alterations could be due to increased levels of cortisol^14^ and/or androgens, oestradiol and beta-endorphins, which cause an alteration in the hypothalamic hypophyseal axis leading to a reduction in the release of luteinising hormone (LH) and the gonadotropin-releasing hormone (GnRH), both involved in the menstrual cycle^7,14,15^. Another consequence of suffering from high anxiety levels seems to be the appearance of eating disorders^16^, particularly in women^2–4,17^. Male and female athletes have a greater risk of suffering from these disorders, in relation to the non-athletic population, especially in sports where a lower weight represents an advantage^3^.

In this work we have analysed the anxiety levels of a population of female paddlers and the possible consequences for their health, such as eating disorders or the regulation of their menstrual cycle.

## Materials and methods

### Informed consent and approval by the Ethics Committee

The protocols for this research have been reviewed and approved by the Ethics Committee of the Universidad Pablo de Olavide with the code 22/3-6 and they respect the fundamental principles of the Declaration of Helsinki, of the Council of Europe’s Convention on Human Rights and Biomedicine, of the UNESCO Universal Declaration on the Human Genome and Human Rights, of the Convention for the Protection of Human Rights and Dignity of the Human Being with regard to the Application of Biology and Medicine (Oviedo Convention on Human Rights and Biomedicine), and also the Spanish law on data protection (Organic Law 3/2018, of 5 December). Informed consent was obtained from all individual participants included in the study. The participant has consented to the submission to the journal.

### Characteristics of the sample

The sample is composed of 47 women, of whom 23 (48.90%) are HLA and 24 are HPA (51.10%), randomly selected among the high-performance sports clubs/centres where they train with similar training loads and identical competition schedules.

The mean age of the HLA is 24.91 years old, which is slightly more than two years over the age of the HPA, 22.83 years old (Table 1). The average weight of the female athletes analysed is 63.62 kg and the average height is 1.68 m, with the mean BMI (22.62 kg/m^2^) within the normal limits for normal weight. The female athletes in this research compete in a wide range of competitions, from championships at regional level or lower to the Olympic Games.

**Table 1:** Characteristics of the sample interval estimate with a 95% confidence level.

	N (%)	Age (years)	Weight (kg)	Height (m)	BMI (Kg/m^2^)
HLA	48.90	24.91 (22.87,26.95)	64.80 (61.68, 67.88)	1.69 (1.66, 1.71)	22.60 (21.86, 22.66)
HPA	51.10	22.83 (21.07, 24.60)	62.50 (59.82, 65.19)	1.66 (1.63, 1.68)	22.64 (21.07, 24.60)
Total/Mean	100	23.85 (22.52, 25.18)	63.62 (61.62, 65.61)	1.68 (1.65, 1.69)	22.62 (22.11, 23.11)

*BMI* body mass index.

### Tests used

#### GAD-7 scale (generalized anxiety disorder)

To detect possible anxiety disorders in the study participants, we used an adapted version of the GAD-7 Assessment in Spanish^18^ obtained from *the Banco de Instrumentos y Metodologías en Salud Mental del Centro de Investigación Biomédica en Red de Salud Mental* (CIBERSAM) (Mental Health Tool and Methodology Bank at the Biomedical Research Centre for the Mental Health Network). Using the score attained, the level of risk of suffering from anxiety is classified according to four categories of severity: minimum risk: from 0 to 4; mild: from 5 to 9; moderate: from 10 to 14; and severe: from 15 to 21.

#### Evaluation of menstrual alterations

The possible menstrual alterations were recorded by means of a questionnaire with seven questions in which information was collected regarding the frequency and duration of menstruation and whether this was affected by training and/or the stage the athletes were at in the season.

#### EAT-26 test (eating attitudes test-26)

Possible eating disorders (ED) were evaluated using the validated Spanish version of the EAT-26 tool^2^. The questions were divided into three subscales:

Dimension 1: diet (13 items regarding behaviours to avoid the consumption of food that causes weight gain or preoccupation with feeling thin).

Dimension 2: bulimia and preoccupation with food (6 items regarding bulimic behaviour and thoughts about food).

Dimension 3: oral control (7 items regarding self-control of dietary intake and possible pressure from others to gain weight).

The evaluation determines the risk of suffering from ED: no risk (0–9); low risk (10–19); and high risk (≥ 20).

### Statistical analysis

We conducted a descriptive study and inferential analysis of the data collected to determine possible significant relationships between the two groups, the HLA and HPA. Where necessary, the variables were assessed to see if they met the underlying hypotheses for normality, using the Shapiro-Wilk test for normality. Levene’s test was used to study the equality of variances. The statistical study of the equality of means for the quantitative variables in both groups (HLA and HPA) was conducted using Student’s t-test for independent samples or the non-parametric Mann-Whitney U test and median test when the hypotheses for normality were not met. To study the possible association between a qualitative variable and the athlete’s level, we chose Pearson’s chi-square test of independence and Fisher’s exact test for 2 × 2 tables. When the requirements for application were not met for Pearson’s chi-square test, categories were grouped together. In all of the contrasts conducted, we set the level of significance at 5% and for the data analysis we used the statistical programme IBM SPSS Statistics v27.

## Results

### Anxiety disorder

All the female paddlers have healthy sleep routines, as 80% sleep for between six and eight hours, with a very small number (2%) sleeping for under six hours. However, as shown in Table 2, the group of less experienced paddlers (HPA) present a higher risk of suffering from an anxiety disorder (9.63) than the HLA (6.64), according to the GAD-7 Assessment. Additionally, it can be confirmed that the difference between groups is moderate, as it is quantified by the effect size studied.

**Table 2:** Study of GAD-7 Scale scores according to the athlete’s level.

	Total score GAD-7 Assessment	SD	P-value (test for normality)	P-value (Levene’s test)	P-value (test for equality of means)	Effect size Cohen’s d
HLA	6.64	2.80	0.61	0.05	0.01	− 0.76 (− 0.75 Hedges’ correction)
HPA	9.63	4.70	0.25			

*SD* standard deviation.

**Figure 1:**
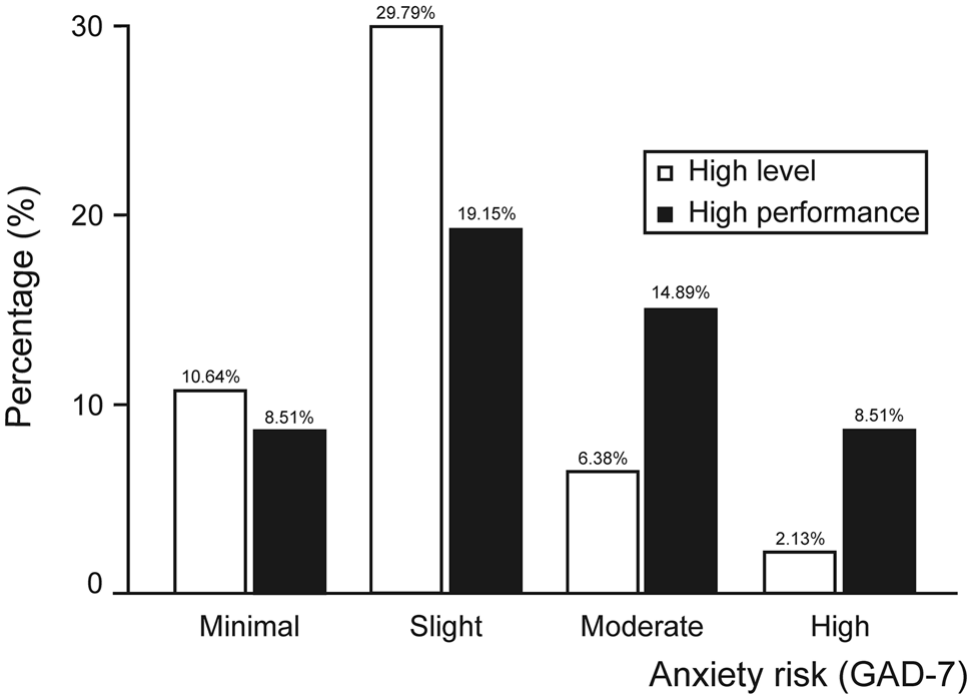
Difference in distribution for the risk of suffering from an anxiety disorder (GAD-7) according to the athlete’s level. Pearson’s chi square test; p-value = 0.037 (5% level of significance).

In Fig. 1 we have categorised the scores from the GAD-7 questionnaire according to the type of risk that the female athlete may suffer from (minimal, mild, moderate or high risk). As can be seen, most of the paddlers in the HLA group present a minimal/mild risk of suffering from anxiety, while the majority of the athletes in the HPA group are in the categories of mild and moderate. It is understood that a third of the population (32%) at this sporting level (HLA/HPA) have a moderate or high risk of suffering from anxiety. These differences are statistically significant (Pearson’s chi square test; p-value = 0.037).

### Study of the paddlers’ menstruation

The mean age for the onset of menstruation is around 13 years old in both groups, which is within the normal range (Table 3). The use of hormonal contraceptives is not habitual, with only 17.10% of the total sample using them, of which 25% are HLA and 75% are HPA.

**Table 3:** Characteristics of menstruation according to type of athlete.

	N	Menarche (years)	Use of hormonal contraceptives (%)	Absence of menstruation in the last 3 months (%)	Modification of menstrual cycle due to an important competition (%)	Modifies training due to menstruation (%)
HLA	23	12.96	25	60	40.91	25
HPA	24	13.21	75	40	59.09	75
Total/Mean	47	13.09	100	100	100	100

In relation to irregularities in menstruation (absence of menstruation for three or more consecutive cycles in the last year) we can observe that 21.30% of the total paddlers have experienced irregular menstruation, of which 60% are HLA and 40% are HPA. However, the most significant result is that 46.80% state that their menstrual cycle is altered when they have an important competition, with these irregularities being more frequent in the HPA group (59.09%) than among the HLA (40.91%). Furthermore, of the female paddlers who adjust their training to their menstruation, 25% are HLA and 75% are HPA.

### Eating disorders

The scores attained on the EAT-26 scale show that there is no significant statistical evidence to confirm that the female athletes in the HLA group present higher risks of suffering from an eating disorder than those in the HPA group or vice versa (see Table 4). Likewise, neither have any significant differences been found in any of the dimensions analysed, as all the p-values arising from all the Mann-Whitney U tests for independent samples and the median tests for independent samples were not significant, both tests conducted on the overall score and for each of the dimensions.

**Table 4:** Mean scores on the EAT-26 scale and their dimensions.

EAT-26 Test score	Category	Mean	Median	SD	Mann–Whitney U test P-value	Median test P-value
Overall	HLA	10.71	6	10.70	ns	ns
	HPA	7.42	4	7.84	ns	ns
Dimension 1	HLA	5.78	3	6.54	ns	ns
	HPA	4.08	1.5	5.02	ns	ns
Dimension 2	HLA	2.26	1	3.10	ns	ns
	HPA	1.45	1	2.06	ns	ns
Dimension 3	HLA	2.13	1	2.98	ns	ns
	HPA	1.87	1	2.65	ns	ns

SD standard deviation, ns test not significant.

p-value > 0.05.

The scores obtained by each athlete on the EAT-26 test also show that 66% of the female athletes do not present a risk of suffering from an eating disorder, 19.1% present a low risk and only 14.9% present a high risk. Furthermore, there is no relationship between the athletes’ level and the risk of suffering from an ED, and both Pearson’s chi-square test (p-value = 0.471) and Fisher’s exact test (0.547) cannot reject the hypothesis for independence of the variables.

### Relationship between the athlete’s level, the level of anxiety and alterations in menstruation

As mentioned above (Table 2), there are significant differences in the GAD-7 anxiety level among the HLA and HPA. These differences can be noted both in the group of women who do not present menstrual alterations (p-value Student’s t-test for independent samples = 0.004) and the group of women who state that they have missed a period for three or more consecutive cycles in the last 12 months (p-value Student’s t-test for independent samples = 0.003), with a great difference in this group of 6.33 absolute percentage points (see Table 5). This also confirms the value of the effect size, indicating a great difference in the group of female athletes with menstrual alterations. Likewise, it is noteworthy that the athletes in the HPA group that definitely present menstrual alterations are on the threshold of presenting a severe level of anxiety (14.50 points in the GAD-7 assessment).

**Table 5:** Relationship between the athlete’s level, level of anxiety and alterations in menstruation.

Alterations in periods over the last 12 months		Total score GAD-7 assessment	SD	P-value (test for normality)	P-value (Levene’s test)	P-value (test for equality of means)	Effect size Cohen’s d
No	HLA	6.06	2.52	0.30	0.12	0.04	− 0.72 (-0.71 Hedges’ correction)
	HPA	8.65	4.23	0.33			
Yes	HLA	8.17	3.19	0.98	0.32	0.03	− 1.76 (− 1.79 Hedges’ correction)
	HPA	14.50	4.20	0.59			

Finally, we checked whether there was statistical evidence to confirm that there was a relationship between having a moderate or high risk of suffering from anxiety and menstrual disorders during three or more cycles during the last year (Fisher’s p-value = 0.042, with a moderately strong relationship being found between these variables; phi coefficient and Cramer’s V = 0.313).

### Relationship between the athlete’s level and the risk of suffering from anxiety

**Figure 2:**
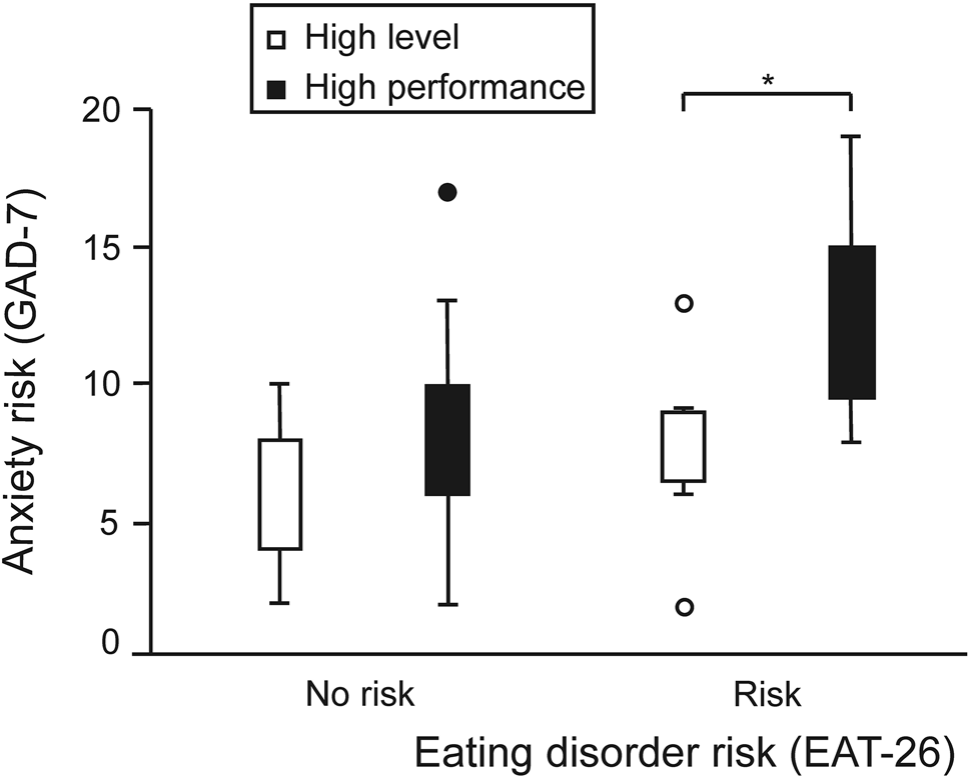
Relationship between the risk of suffering from anxiety and the risk of suffering from an ED according to the level of training. *, Student’s t-test p-value for independent samples = 0.038.

If we take the female athletes who present a high risk of suffering from possible eating disorders as a reference, there is a statistically significant difference (p = 0.038) in the risk of suffering from anxiety between the HLA and the HPA, with the latter showing higher levels of anxiety (Fig. 2).

#### Relationship between the athlete’s level, hours of training and the risk of suffering from anxiety

**Figure 3:**
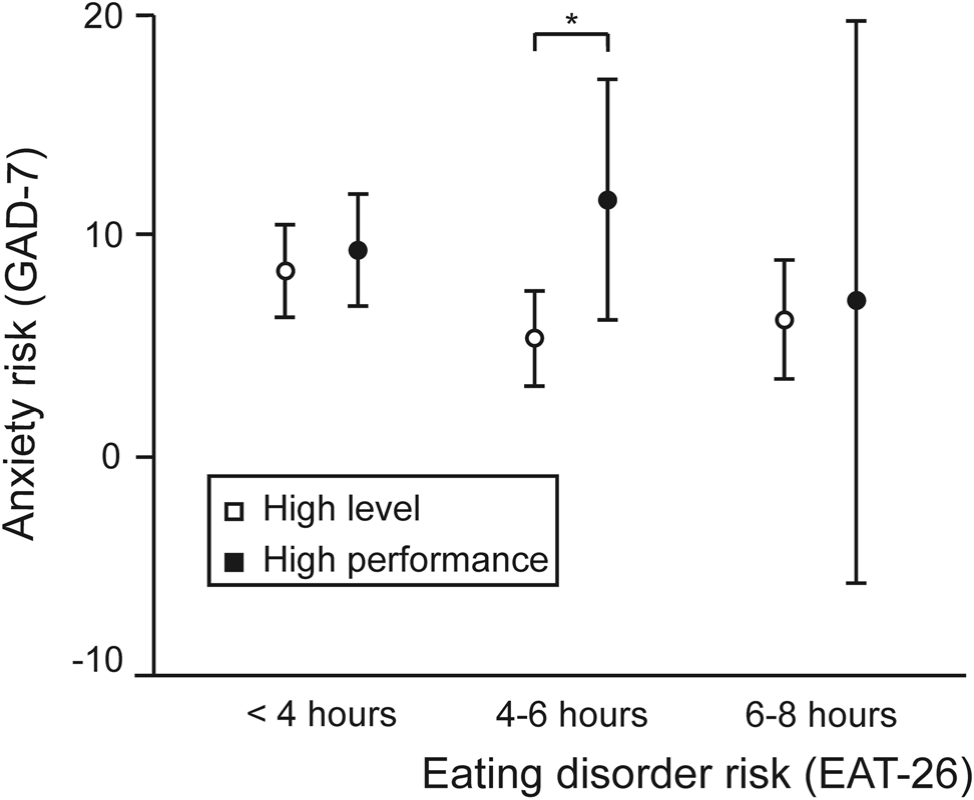
Relationship between the risk of suffering from anxiety and the number of hours spent training per day according to the sporting level. *, Student’s t-test p-value for independent samples = 0.006.

Finally, significant differences were found between the risk of suffering from anxiety and the athlete’s level, in the group that trains between four and six hours a day (Fig. 3): the women in the HPA group present almost double the levels of anxiety (11.60) than their HLA fellows (5.33). This group, the HPA, are those who, apart from their sporting career, are trying to develop a parallel activity to training, normally an academic path: the dual career.

## Discussion

Mental disorders caused by anxiety have been on the rise recently among the general population. Extrapolating these data, and using other studies published as a basis^7–10,19^, suggests that there has also been an increase among athletes. In view of this, our results demonstrate that the majority of female paddlers present a certain risk of suffering from anxiety. Furthermore, we have also observed higher levels of anxiety among the female athletes at a lower level (HPA). These results coincide with others that have already been published, in which the symptoms of anxiety were less significant among more experienced female athletes^6,20^ and they are also in line with the study by Alonso^1^, where the female sex, a lower age and having less sporting experience are associated with greater competitive anxiety in athletes^1^.

Some authors link a moderate or high risk of suffering from anxiety^7,8^ to menstrual alterations (absence of periods or longer periods than usual). Although only 21.30% out of the total of our female paddlers suffered from amenorrhea in the last three months, 60% of them were in the HLA group. This could be due to the level of the competitions they face or even to the use of oral contraceptives. These alterations in the menstrual cycle usually coincide, moreover, with important competitions, and are greater among the HPA population than among the HLA.

The relationship between high levels of anxiety and eating disorders is not so clear. Although some authors claim it exists^2–4,16,17^, we have not found proof of this relationship in our samples of athletes, which coincides with other works suggesting that participation in some type of physical activity can have positive psychological effects^21^.

In terms of perspectives, the results of this work provide a great deal of information about the state of the mental and physical health of our female athletes in Spain. Although awareness is growing regarding the effects on health of exercising at a professional level^3,6–10,13,17,21^, it is important to define and update these data to be able to act on and prevent possible mental and physical health problems, which are increasingly frequent among the population, particularly among females, so that suitable interventions can be developed.

## Conclusions

According to our results, elite Spanish female paddlers present a risk of suffering from anxiety disorders. Among these athletes, the least experienced have a greater chance of suffering from these disorders, probably due to the dual career that these athletes usually undertake. The anxiety levels recorded usually appear somatically as alterations in menstruation. Additionally, we should monitor less-experienced female athletes with higher anxiety levels, as they have also a greater risk of suffering from eating disorders. The specific conclusions are that a third of the population analysed presents a moderate/high risk of suffering from anxiety and less-experienced female athletes (HPA) are that have a greater risk of suffering from eating disorders. Moreover, the female athletes in the HLA group suffer from more irregularities in menstruation than those in the HPA group.

It stands out that almost half the total female paddlers suffer from menstrual alterations before an important competition, with this number rising among athletes at HPA level, because of that three out of every four athletes at HPA level adjust their training to their menstrual cycle. In summary, it is important to mention that we have found a positive relationship between a moderate or high risk of suffering from anxiety and menstrual disorders during three or more cycles during the last year and although we have not found a relationship between the level of training and the risk of suffering from eating disorders, it is necessary to emphasize that female athletes at HPA level who showed higher anxiety levels also presented a greater risk of suffering from eating disorders. Finally, it is essential to control HPA level athletes who train between four and six hours a day as they show anxiety levels that double those of more experienced female athletes (HLA).

## Practical applications

This study shows that in female paddlers, anxiety is somatized in menstrual disorders and / or eating disorders so it is necessary to show coaches the need to adapt the load of training to the menstrual cycles of their paddlers.

It is also important that athletes are supervised by a multidisciplinary team, where in addition to the coach, physiotherapist or nutritionist, there is also a sports psychologist to help athletes to work on anxiety and focus on their performance regardless of the result. This multidisciplinary team should be present from the lower categories since, as our results indicate, it is the youngest and most inexperienced paddlers who are more sensitive to having menstrual or eating disorders due to anxiety.

## Data Availability

The datasets generated during and/or analysed during the current study are available from the corresponding author on reasonable request.
